# The role of melanopsin photoreception on visual attention linked pupil responses

**DOI:** 10.1111/ejn.15659

**Published:** 2022-04-07

**Authors:** Subodh Gnyawali, Beatrix Feigl, Prakash Adhikari, Andrew J. Zele

**Affiliations:** ^1^ Melanopsin Photoreception and Visual Science Laboratories, Centre for Vision and Eye Research Queensland University of Technology (QUT) Brisbane QLD Australia; ^2^ School of Biomedical Sciences Queensland University of Technology (QUT) Brisbane QLD Australia; ^3^ Queensland Eye Institute Brisbane QLD Australia; ^4^ School of Optometry and Vision Science Queensland University of Technology (QUT) Brisbane QLD Australia

**Keywords:** decision‐making, melanopsin, phasic arousal, reaction time, task‐evoked pupil dilation, visual attention

## Abstract

A decision during a visual task is marked by a task‐evoked pupil dilation (TEPD) that is linked to the global cortical arousal state. Melanopsin expressing intrinsically photosensitive retinal ganglion cells (ipRGCs) form the afferent pathway for this pupil response. Melanopsin activation also influences mood and arousal and increases activity in decision‐making brain areas that receive direct ipRGC projections. Here, an optical photostimulation method controlled the excitations of all five photoreceptor classes in the human eye to isolate melanopsin‐mediated photoreception. We hypothesised that the TEPD can be driven by directing active visual covert attention through the ipRGC pathway. When observers are completely certain of the stimulus presence, melanopsin‐directed stimulation produces a TEPD of similar amplitude to a cone‐directed stimulation, with their combination producing larger amplitudes. This dilation is satisfactorily modelled by linear addition with a higher melanopsin weighting in ipRGCs. Visual reaction times were longest in response to melanopsin‐directed lights. Next, we asked whether the afferent photoreceptor input and decision certainty, controlled by priming the observer's a priori expectation, interact to drive the TEPD. Signal detection analysis showed that by fixing the predecision certainty (bias), the phasic arousal and TEPD amplitude vary with observer criterion (c′) and sensitivity (d′) but not with preferential activation of melanopsin. The signature feature of the melanopsin response during attention was a biphasic TEPD. We conclude that active covert attention can be modulated by visual information mediated via ipRGCs, but that phasic arousal responses marked using the TEPD are not increased by higher levels of melanopsin activation.

## Graphical abstract

Melanopsin expressing intrinsically photosensitive retinal ganglion cells (ipRGCs) mediate visual information during active covert attention. A biphasic task‐evoked pupil dilation (TEPD) is the signature marker of melanopsin‐directed phasic arousal. When decision uncertainty is high, the amplitude and timing of the TEPD are independent of the level of melanopsin stimulation in the light.

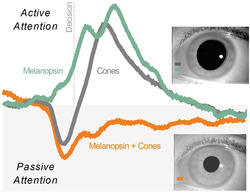

## 1 INTRODUCTION

The amplitude and timing of the visual task‐evoked pupil dilation (TEPD), a marker of decision‐making (Einhäuser, 2017; Larsen & Waters, 2018), are influenced by contrast‐based saliency (Wang & Munoz, 2014), task complexity (Moresi et al., 2008), and the level of observer uncertainty (Colizoli et al., 2018; de Gee et al., 2014; Urai et al., 2017). This dilation as a proxy measure of the global cortical arousal state (McGinley et al., 2015) is termed phasic arousal (de Gee et al., 2017). It is associated with increased noradrenergic locus coeruleus activity (Aston‐Jones & Cohen, 2005; Foote et al., 1983) through inhibition of parasympathetic inputs to the Edinger–Westphal nucleus that innervates the iris sphincter muscle for pupil constriction (Beatty & Lucero‐Wagoner, 2000). Intrinsically photosensitive retinal ganglion cells (ipRGCs) form the primary afferent pathway driving the pupil light reflex in nonhuman primates (Hannibal et al., 2014; Ostrin et al., 2018) by integrating the extrinsic cone‐ and rod‐initiated responses (Dacey et al., 2005) as well as the intrinsic melanopsin photoresponse in humans (Gamlin et al., 2007; Markwell et al., 2010), but their role in the TEPD is yet to be determined. In rodents, the light‐induced enhancement of mood and arousal is also known to be mediated via the melanopsin pathway (LeGates et al., 2014). In humans, the exposure to melanopsin‐enriched lights (e.g., chromatic and bluish/cyan appearing) causes increased activity in brain regions involved in decision‐making, including the amygdala, pulvinar, and superior colliculus (Chellappa et al., 2014; Vandewalle et al., 2007) and improves subjective alertness and work performance (Chellappa et al., 2011; Viola et al., 2008). Such melanopsin‐enriched lights are not optimal for characterising the relative melanopsin and cone photoreceptor contributions to brain regions involved in decision‐making because they are nonselective in their activation of the melanopsin and cone inputs to ipRGCs and/or the conventional retinogeniculate pathways. By means of experimental conditions designed to drive the intrinsic melanopsin photoresponse independently of that from the cone and rod photoreceptors, we characterise the effect that changes in an observer's covert attentional state have on phasic arousal quantified by the TEPD when sensory inputs are transmitted via specific afferent pathways. Given that melanopsin‐enriched lights enhance the activity of decision‐related brain areas that receive direct ipRGC projections (Chellappa et al., 2014; Vandewalle et al., 2007), we hypothesise that intrinsic melanopsin photoresponses will drive a TEPD of different amplitude and timing than in response to melanopsin‐silenced, cone‐directed lights. We reveal how the pupil control pathway combines the different photoreceptor inputs during active and passive covert attention tasks.

At any moment, the pupil diameter reflects the consequence of changes in complex neurological pathways to somatic and autonomic activity (Zele & Gamlin, 2020). The TEPD amplitude also varies from trial to trial (de Gee et al., 2014). Post‐hoc analyses of the between‐trial TEPD variability suggest that higher amplitudes relate to lower decision bias (criterion c′) and the higher phasic arousal positively correlates with locus coeruleus activity (de Gee et al., 2017). Those post‐hoc analyses were however, of data obtained using paradigms during which predecision intrinsic bias was free to vary within‐ and between‐observers during the experiment. We therefore separate the effect of decision uncertainty on pupil linked arousal from the photoreceptor‐directed contribution to the TEPD by preferentially biasing the observer's predecision judgement. The effect of the observer criterion (c′) and sensitivity (d′) on the TEPD was then analysed according to signal detection theory (SDT). We hypothesise that by reducing observer bias through priming, the higher phasic arousal will result in larger TEPD amplitudes during a discrimination task, independent of the preinformed stimulus likelihood or the melanopsin level in the light.

## 2 MATERIALS AND METHODS

### 2.1 Participants

All participants (*n* = 6, age range 21–41 years, three males, three females) had trichromatic colour vision (Rayleigh match, pseudoisochromatic plates, D‐15), best‐corrected distance visual acuity better than 0.0 (Bailey Lovie Log MAR; equivalent to 6/6 Snellen), lens opacification < N2‐2, P1, C2 (LOCS II) and no ocular pathology as confirmed with funduscopy (Beta 200 LED Ophthalmoscope, Heine Optotechnik, Germany), fundus photography (Canon Non Mydriatic retinal camera, CR‐DGi, Canon Inc.) and optical coherence topography (RS‐3000 Advance, HD OCT, Nidek, USA). One participant was an author, and the other five participants were naïve to the purpose of the study; there were no differences in the response patterns of the naïve and experienced observers. The experiments were approved by the Human Research Ethics Committee at Queensland University of Technology (approval no. 1700000510) and adhered to the tenets of the declaration of Helsinki. Participants provided written informed consent. In addition to the individual observer calibrations required to generate and confirm the photoreceptor‐directed stimulation (details below), each participant was tested for a total of ~2400 trials, and so we applied within‐subject statistical analysis of the data.

### 2.2 Apparatus and calibrations

The excitations of melanopsin, rhodopsin and three‐cone opsins were independently controlled by silent substitution (Estevez & Spekreijse, 1982; Shapiro et al., 1996) using a custom‐built 5‐primary photostimulator (Cao et al., 2015) with light emitting diode and interference filter combinations (Ealing, Natick, MA, USA) producing five narrowband primary lights having peak wavelengths (± full width at half maximum) at 456 nm (10 nm), 488 nm (11 nm), 540 nm (10 nm), 594 nm (14 nm), and 633 nm (15 nm). The light outputs were controlled by an Arduino based system (Arduino Uno SMDR3, Model A000073) using a custom‐built software (Xcode 3.2.3, 64‐bit, Apple, Inc., Cupertino, CA, USA) (Cao et al., 2015; Zele et al., 2018, 2019a). The photostimulator was calibrated periodically during the experiments; spectral outputs of the primary lights were measured with a spectroradiometer (StellarNet, Tampa, FL, USA) and luminance outputs were measured with an ILT1700 Research Radiometer (International Light Technologies, Inc., Peabody, MA, USA).

**FIGURE 1:**
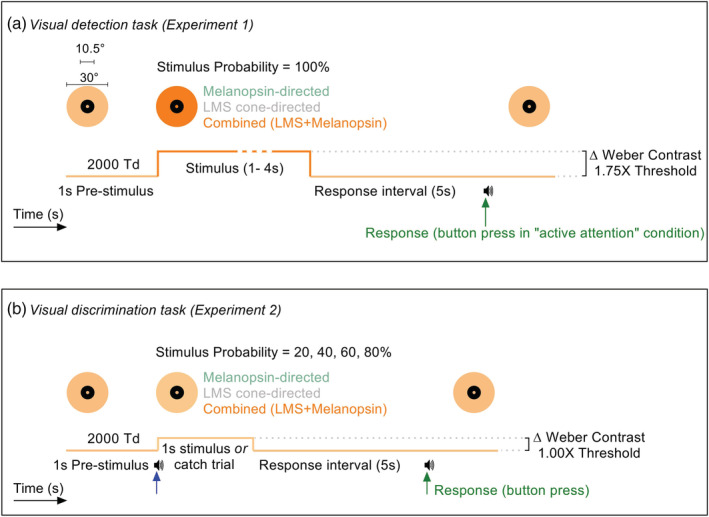
Schematics of the spatio‐temporal properties of the stimulus paradigms for the active and passive covert attention conditions in the visual detection and discrimination tasks. (a) For the visual detection task (Experiment 1), the three photoreceptor‐directed stimuli were contrast scaled to 1.75× the individual detection threshold and presented at 100% probability. During the passive attention condition, participants focussed on the fixation spot within the centre of the 10.5° diameter macular blocker and no response was necessary during a trial. During the active attention condition, participants were required to maintain focus on the fixation marker and tasked with responding to stimulus offset by pressing a button on a hand‐held game pad (within 1.5 s of the 5 s post‐stimulus response interval). A short audio beep (<50 ms, ~440 Hz) at the button press (green upward arrow) indicated successful completion of the trial and signalled the observer to prepare for a successive trial. The prestimulus duration was fixed (1 s) and the stimulus duration was randomly varied between 1 and 4 s (1 s steps). The photoreceptor excitations and stimulus durations were randomised in separate blocks across the testing sessions in the attention conditions. (b) For the visual discrimination task (Experiment 2), the three photoreceptor‐directed stimuli (1 s duration) were contrast scaled to 1.00× the individual detection threshold and presented at four probabilities (20, 40, 60 and 80%) using a block design, each with 20 trials. In each trial, the observers task was to discriminate between the stimulus offset and the catch trial (no‐stimulus) using a button‐press. A short audio beep at stimulus onset (blue arrow) prepared the observer for their response. A button press (green arrow) recorded their decision and signalled an audio beep to indicate successful trial completion. Consensual pupil responses were continuously recorded

The stimuli were presented to the right eye in Maxwellian view through a 2 mm artificial pupil. The stimulus was an annular field of 30° outer diameter with a central 10.5° diameter black macular blocker; the observers were instructed to fixate on a small marker (<1 min arc) positioned in the centre of the macular blocker (Figure 1). An orange appearing adaptation field (CIE x = 0.549, y = 0.410) at 2000 photopic Td with relative photoreceptor excitations for L‐cone = 0.752, M‐cone = 0.248, S‐cone = 0.105, Rod = 0.319 and Melanopsin = 0.235 was chosen to maximise the instrument gamut. Three photoreceptor excitation conditions were generated: (1) melanopsin‐directed incremental stimuli with no change in the excitation of rhodopsin and three cone opsins (i.e., a melanopsin‐directed stimulus); (2) in phase modulated L‐, M‐, and S‐cones to produce cone luminance‐ (+L + M + S) directed incremental stimuli with no change in the excitation of rods and melanopsin (i.e., cone‐directed stimuli); and (3) an additive mixture of melanopsin and LMS cone stimuli with no change in the rhodopsin excitation (i.e., combined LMS + Melanopsin stimuli).

Individual differences in prereceptoral filtering brought about by lenticular attenuation (Pokorny et al., 2004; Sun et al., 2001) and photopigment polymorphisms (Jacobs, 1996) were compensated for by an individual observer calibration measuring the sensitivity differences for different combinations of the primary lights between the participant and CIE 1964 10° standard observer using Heterochromatic Flicker Photometry (HFP) (Adhikari et al., 2019; Zele et al., 2018, 2019b) to ensure equiluminance across the photoreceptor stimulation conditions (Uprety et al., 2021). The precision of the silent substitution for isolating each photoreceptor class was verified in each observer using a series of psychophysical tests, including bleach recovery of rod‐ and cone‐directed visual thresholds, photoreceptor‐directed visual thresholds measured under scotopic, mesopic, and photopic light adaptation, and cone‐directed colour matches to rod or melanopsin‐directed stimuli. Details of the test procedures are described elsewhere (Cao et al., 2005, 2008; Zele et al., 2018, 2019b).

### 2.3 Determination of threshold

Stimulus contrast was scaled in multiples of the observer threshold (1.00× or 1.75× Threshold Units, TU) measured separately with each of the three photoreceptor‐directed test stimuli. In Experiment 1 (detection task), the stimuli were supra‐threshold and therefore had high salience (1.75× TU). In Experiment 2 (discrimination task), the stimuli were set to threshold and therefore had low salience (1.00× TU). Contrast detection thresholds were determined using a two‐yes‐one‐no double alternating staircase procedure (Zele et al., 2006). The initial stimulus contrasts of the two staircases were supra‐threshold and offset by 10% Weber contrast. Detection threshold was calculated as the average of the last six reversals for each staircase. Given that detection thresholds for melanopsin‐directed stimuli are higher than for cone luminance‐directed stimuli (Zele et al., 2018), the more sensitive cone‐luminance process (L + M + S‐cones) would drive detection if the two thresholds contrast were simply added to generate a combination stimulus. Therefore, the LMS and melanopsin contrasts in the combination stimulus were initially set at their respective threshold levels and the combination threshold was estimated using a method of constant stimuli (MOCS). The MOCS procedure included six contrast combinations spanning the threshold range in 0.2% increments, each presented 50 times. In the MOCS, the combination stimulus with the lowest contrast had the LMS cone contrast set at half its measured detection threshold to allow for any melanopsin enhancement of cone‐mediated vision (Zele et al., 2019b), with the melanopsin contrast set at the LMS:melanopsin contrast ratio. The detection threshold for the LMS + melanopsin combination stimulus was defined at 63.21% correct responses (Gilchrist et al., 2005) on the best‐fitting Weibull function of the frequency of seeing plotted as a function of contrast ratio. For each observer, the LMS:melanopsin combination contrast detection threshold ratio was computed separately and returned an average contrast ratio of 1:1.19.

Detection thresholds for all photoreceptor‐directed stimuli were re‐measured regularly as the experiment progressed and updated as required. This procedure limited any dependence of the outcome measures (TEPD and reaction time [RT]) on variations in the observer threshold over time (Zele et al., 2007). The Weber contrast thresholds were on average (μ ± SEM; *n* = 6 observers) 9.7 ± 1.6% for melanopsin‐directed, 5.7 ± 0.3% for cone‐luminance and [(3.5 ± 0.7%) + (5.46 ± 0.65%)] for combined cone and melanopsin‐directed stimuli.

### 2.4 Experimental design

The detection (Experiment 1) and discrimination (Experiment 2) experiments were designed to differentiate the effects of the melanopsin and/or cone‐opsin inputs to the TEPD following changes in selective covert attention to temporal offset of a spatially constant annular stimulus. The detection task set the stimulus contrasts at 1.75× threshold; the discrimination task set the stimulus contrasts to threshold. During detection tasks (Figure 1a, Experiment 1), the observer covertly attends to high salience, supra‐threshold stimuli and actively responds to stimulus offset with a button press (i.e., endogenous attention); in the control condition, the observer attends passively to the supra‐threshold stimulus without providing a response (i.e., exogenous/transient attention). Given that attention can increase the response of neural populations tuned to a feature within an active condition (Alais & Blake, 1999), we examined whether direct projections of ipRGCs to arousal centres (Chellappa et al., 2014; Vandewalle et al., 2007) lead to larger melanopsin‐directed TEPD responses than in the cone‐directed tasks. Stimuli were presented at 100% probability and anticipatory responses to stimulus offset were minimised by implementing four stimulus durations (1000 to 4000 ms in 1000 ms steps) that were randomly interleaved in separate trial blocks. The stimulus durations were chosen to increase the melanopsin‐driven pupil response amplitudes (Adhikari et al., 2015; Kelbsch et al., 2019; Park et al., 2011) and because they are beneficial in minimising penumbral cone intrusion during steady‐state light adaptation (Spitschan et al., 2015; Yamakawa et al., 2019).

During discrimination tasks (Figure 1b, Experiment 2) with threshold level, low salience stimuli, the observer's a priori expectation was primed by preinforming of the stimulus probability; either 20%, 40%, 60%, or 80% of the trials included the stimulus. The observer was tasked with correctly responding to the stimulus presence with a button press (“yes” or “no” response). Stimulus onset was signalled by a brief auditory tone (50 ms, ~440 Hz) to alert the observer to prepare for their response but was spaced long enough from stimulus offset (1000 ms) to limit the integration of auditory and visual signals that can influence the visual task‐evoked pupil response and RT (Adam et al., 2014; Hershenson, 1962; Jahanshahi et al., 1992). We informed participants of the relative event frequency at the beginning of a session to introduce a bias in the preparatory process and response execution (Friedman et al., 1973; Reinhard et al., 2007) by allowing the planning of motor responses during the prestimulus period based on their expectation that the probability of their choice was correct (Gehring et al., 1992). The TEPD and visual choice RT during the discrimination task is therefore modulated by the level of decision uncertainty. We then estimated the decision bias utilising signal detection theory (SDT) based on the hit and false‐alarm rates (de Gee et al., 2014; Green & Swets, 1966; Macmillan & Creelman, 2004), with the advantage that the observer's intrinsic bias is exhibited in the outcome because their belief state is fixed (Kiani et al., 2014). All task‐evoked pupil responses and visual RT data from the discrimination task were then analysed according to the four trial types: hits (H), misses (M), correct rejects (CR), and false alarms (FA) (Macmillan & Creelman, 2004). The probability of a stimulus presentation occurring in a testing session (with 20 trials) is defined as the stimulus probability for hits and catch (1‐stimulus probability) for correct rejects (Figure 3). When combining the stimulus and nonstimulus related outcomes, the probability of both the stimulus and nonstimulus (catch) is defined as the target probability (Figure 4). We could determine if an observer changed their response criterion during the experiment (e.g., attending to the “no” stimulus in the low stimulus probability condition rather than to the “yes” stimulus) if the pattern of TEPD amplitudes were similar for the low and high stimulus probability events (i.e., a U shaped‐pattern as a function of stimulus probability); there was no evidence in the data for this response pattern.

#### 2.4.1 TEPD

Pupil diameters from the consensual pupil response were measured continuously during all trials at a 60‐Hz sampling rate using an infra‐red (*λ*_max_ = 851 nm) video camera (640 × 480 pixels; 60 Hz; Point Gray FMVU‐03MTM‐CS; Richmond, BC, Canada; Computar TEC 55 mm telecentric lens; Computar, Cary, NC, USA) with the full pupil area perpendicular to the camera's line of sight. The pupil diameter was used in the analysis in accordance with our laboratory procedures (Feigl et al., 2011; Kelbsch et al., 2019). Data processing was conducted with the researcher blind to the stimulation conditions and the observer responses. Pupil responses were analysed with reference to the baseline pupil diameter on each trial (average of the 500‐ms prestimulus resting pupil data). Each trial was normalised to its own baseline pupil size to limit the effect of fluctuations and control for individual differences in pupil diameter. The pupil response metric during stimulus presentation was the time to the peak pupil constriction amplitude (maximum constriction, % baseline). Post‐stimulus pupil response metrics included the TEPD amplitude (% baseline) and the maximum (peak) post‐stimulus dilation amplitude, quantified between zero and 1500 ms from the subjective response (i.e., RT) in the active covert attention conditions, and from stimulus offset in the passive attention conditions.

The researcher monitored the observer fixation in real‐time during each session. Post‐hoc analysis excluded trials with fixation losses and gaze deviations more than half the stimulus annulus size (15° visual angle) or persisting during more than 10% of the trial time window (Adhikari et al., 2015; de Gee et al., 2014). Trials with 10% or more missing data due to blinks or lid artefacts were also excluded from the analysis; so was the first trial in a session, trials with blinks or fixation losses occurring between 500 ms before stimulus onset and 1500 ms after the decision interval (de Gee et al., 2014), and trials with peak pupil constriction and dilation amplitudes beyond ± 2.5 standard deviations (99.38%) from the means.

#### 2.4.2 RT

Visual RTs in both the detection and discrimination tasks were defined as the time (ms) of the participant's subjective response (button press) to the offset of the photoreceptor‐directed stimulus or a catch trial (no‐stimulus). A fixed fore‐period (1000 ms) maintained constant light adaptation during each trial (Cao et al., 2007). Detection RTs were reported by a single button press whereas discrimination RTs were reported either as a “yes” or “no” by pressing one of the two assigned buttons on a hand‐held game pad. The RT was referenced to the CPU log time and calculated using a custom written script (MATLAB, version R 2016b, Mathworks, USA). Trials with anticipatory (RT < 200 ms) or missed responses (RT > 1500 ms) and trials with RTs greater than ±2.5 standard deviations from the mean were discarded (Cao et al., 2007). For all analyses, the pupil response on each trial was time locked to its RT to eliminate the effect of time focussed response preparation (Jennings & van der Molen, 2005; Moresi et al., 2008) and RT variability (Richer et al., 1983) on TEPD. An optimal bin width for the RT distribution was calculated based on the sample size and standard deviation (Cao & Pokorny, 2010; Scott, 1979) for each photoreceptor‐excitation condition and the distribution described by the best‐fitting hyperbolic secant function.

#### 2.4.3 General procedure

During the experiments, participants were comfortably seated on an adjustable chair, were explained the testing procedures, then aligned in Maxwellian view in the 5‐primary photostimulator. Testing was conducted in a darkened (<1 lux) and sound attenuated laboratory to control for the effect of ambient illumination and auditory noise on pupil responses. Testing sessions were generally completed during the mornings to benefit from higher alertness levels (Maierova et al., 2016) and to minimise the effect of circadian variation on pupil responses mediated by melanopsin ganglion cells (Zele et al., 2011). The testing sessions were restricted to <1 h duration to minimise the effect of observer fatigue, sleepiness, and attention loss. Observers could take breaks when required during the sessions. All measurements started after 10 min of dark adaptation followed by 2 min of background light adaptation. Each observer completed at least 3 practice sessions to familiarise themselves with the testing protocols. Data from the practice sessions were not included in the analysis.

### 2.5 Statistical analysis

The global average of ~100 trials per stimulus condition per observer was calculated and the variability expressed as mean ± standard error of the mean (SEM). Each observer was tested for ~1200 total trials in each of the detection or discrimination task (3 photoreceptor type‐directed stimulus × 4 stimulus duration/probability combination x ~ 100 trials per stimulus condition) to obtain a sufficient sampling distribution (~2400 trials in total). After all the exclusion criteria (missing data, blinks, fixation loss at stimulus onset and decision‐making interval, very high/low amplitudes of constriction/dilation; >2.5 SD from the mean [anticipatory/missed responses]) were applied on the trials, ~28% of the trials in detection task and ~31% of the trials in discrimination task were discarded which is, as expected, higher than reported in studies using distinct spatial features and larger energy gradients in their stimuli (de Gee et al., 2014; Urai et al., 2017). A repeated measures factorial‐ANOVA with Bonferroni adjustment was applied on normally distributed (Shapiro–Wilk test) and homogenous (Levene's test) pupil data to determine the effect of the following on TEPD and visual choice RT: (1) the photoreceptor‐directed stimulation and stimulus duration in the detection task and (2) the photoreceptor‐directed stimulation and observer uncertainty in the discrimination task. We report all main effects, statistically significant interactions, and the multiple comparisons. All statistical analyses were conducted using SPSS software (version 25, IBM Corp, USA) and figures were generated using GraphPad Prism (v8.2.1; GraphPad Software, San Diego, California, USA). Any comparisons with a *p* value <0.05 were considered statistically significant, and those with marginal or no significance include the effect sizes described using partial eta‐squared (*η*_p_^2^) values. A large effect size was considered for *η*_p_^2^ > 0.25 and small effect size for *η*_p_^2^ < 0.01.

## 3 RESULTS

### 3.1 Melanopsin drives the TEPD during active covert attention

**FIGURE 2:**
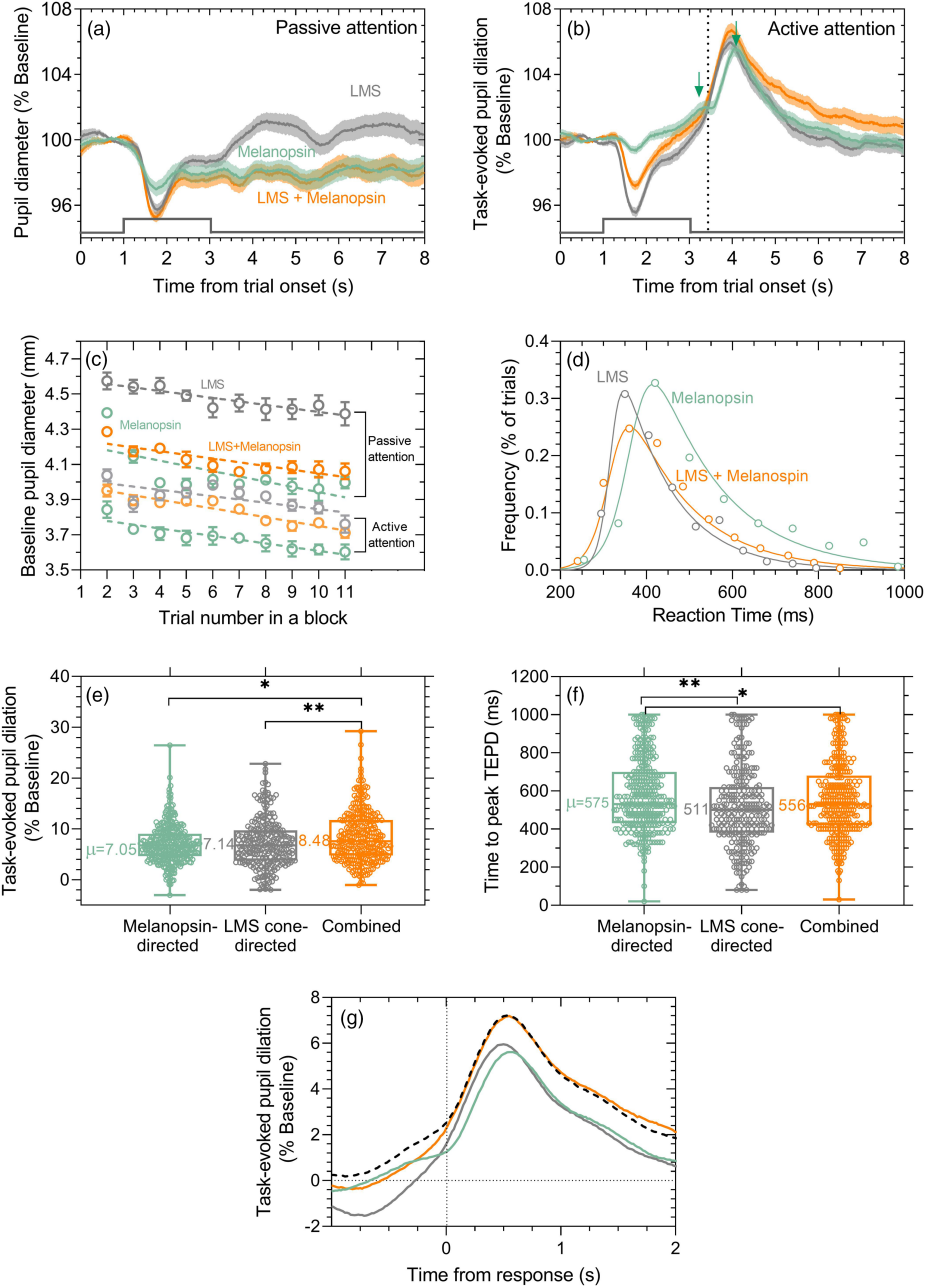
Photoreceptor‐directed pupil responses and reaction times (RT) during active and passive attention in a visual detection task. Data (*n* = 6 observers) are coded green for melanopsin‐directed responses, grey for LMS‐cone‐directed responses and orange for the combined‐stimulus responses. (a) Pupil responses to 1 s duration incremental pulses of three photoreceptor‐directed stimulus types during passive attention. Data show the average ± 95% confidence limits of ~600 total trials per condition. (b) Task‐evoked pupil dilation (TEPD) during active covert attention (average ± 95% confidence limits). The vertical dotted line approximates the average time of the observer response (reaction time) and the green down‐arrows denote the timing of the peaks of the biphasic dilation in response to melanopsin‐containing stimuli. (c) Average baseline diameter (mm ± SEM) across all observers and repeats as a function of the trial number during testing blocks with passive attention or active attention (open circular symbols). (d) Visual reaction time frequency distributions to offset of the supra‐threshold (1.75× threshold) photoreceptor‐directed stimuli are described by their best‐fitting hyperbolic secant functions (solid lines). Data were pooled across all observers and trials. (e) Boxplots (median, interquartile range and whiskers for the minimum and maximum) of the RT‐locked TEPD amplitudes (% baseline) for all observers and trials for the three photoreceptor‐directed stimuli. The group average TEPD amplitude is written. (f) Boxplots of the RT‐locked time to peak TEPD amplitude for all observers and trials for the three photoreceptor‐directed stimuli. The group average time to peak TEPD amplitudes is written. (g) Predictive model (black dashed line, Equation 1) of the RT‐locked (time zero) TEPD with a weighted linear addition of the measured melanopsin‐ (green) and cone‐directed inputs (grey) to the pupil control pathway provides an adequate description of the measured combined (melanopsin and cone) pupil response (orange). Asterisks represent *p < 0.05, **p < 0.001

To determine the relative weights of the photoreceptor‐directed inputs to the TEPD, we compared the TEPD amplitude and timing in response to detection of supra‐threshold melanopsin‐directed, cone‐directed, and their combination (Figure 1a) in passive and active attention conditions (Figure 2a,b). We initially controlled for the decrease in pupil diameter over‐time by normalising the TEPD measured during each trial to its own prestimulus baseline pupil size (Figure 2c). With passive attention, the pupil constricted to stimulus onset and the melanopsin‐directed and combined stimuli both elicited the characteristic sustained post‐illumination pupil response (PIPR) while the cone‐directed pupil response rapidly re‐dilated to baseline following stimulus offset (Figure 2a). Active attention caused a TEPD amplitude (range: 5.8–7.9% > baseline) that peaked on average between 460 and 735 ms after the observer's response for all stimuli (Figure 2b). Compared with the passive attention control condition, the active covert attention to melanopsin‐directed stimuli attenuated the predecision peak pupil constriction amplitudes by ~50% whereas cone‐directed predecision peak pupil constriction amplitudes increased by ~9% (Figure 2b). Compared with the passive attention control condition, the active covert attention to melanopsin‐directed stimuli increased the post‐decision TEPD amplitudes by ~7% whereas cone‐directed post‐decision TEPD amplitudes increased by ~4% (Figure 2b). Actively attending to stimulus offset also decreased the prestimulus baseline pupil diameter with no effect of stimulus duration and a significant main effect for the photoreceptor‐directed stimulation (RMF‐ANOVA *F*_2,510_ = 25.36, *p* = 0.000, *η*_p_^2^ = 0.09), with the smallest pupil diameters for melanopsin‐directed stimulation (Figure 2c).

### 3.2 Visual RTs are longest in response to melanopsin‐directed photostimulation

The RT analysis establishes the difference in the speeded responses mediated via the melanopsin and cone pathways and forms the time‐locked reference marker for the TEPD (Figure 2d). Visual RTs were independent of stimulus duration, ruling out any effect of response anticipation but showed a significant main effect for photoreceptor‐directed stimulation (RMF‐ANOVA *F*_2,516_ = 69.37, *p* = 0.000, *η*_p_^2^ = 0.21) with melanopsin‐directed RTs (575.3 ± 15.3 ms; mean ± SEM) significantly longer than with cone‐directed (Bonferroni's: 116.76 ± 10.94 ms, *p* = 0.000) and combination stimuli (Bonferroni's: 92.32 ± 11.78 ms, *p* = 0.000). There were no significant differences between the RTs for cone‐directed and combination stimuli (Bonferroni's: 24.44 ± 8.35 ms, *p* = 0.06).

### 3.3 Melanopsin activation during active covert attention causes a slow TEPD that combines additively with cone‐signals in ipRGCs

As cone‐mediated RTs are inversely related to the amplitude and timing of the TEPD (Richer et al., 1983), we time‐locked all pupil traces to their respective RTs to control for the possibility that longer melanopsin RTs could artefactually lead to smaller and delayed TEPD amplitudes. These RT‐locked TEPD amplitudes (Figure 2e) were independent of stimulus duration but showed a significant main effect for photoreceptor‐directed stimulation (RMF‐ANOVA, *F*_2,524_ = 8.31, *p* = 0.000, *η*_p_^2^ = 0.03) with combination TEPD amplitudes significantly larger than melanopsin‐directed (Bonferroni's: 1.16 ± 0.41%, *p* = 0.02) or cone‐directed stimuli (Bonferroni's = 1.52 ± 0.38%, *p* = 0.000). The melanopsin‐ and cone‐mediated TEPD amplitudes, however, were not significantly different (Bonferroni's: 0.36 ± 0.38%, *p* = 1.00) (Figure 2e) which indicates that the TEPD amplification is independent of the opsin so does not depend on the cortical projection of the afferent photoreceptor signal.

The time to peak TEPD (Figure 2f) was independent of stimulus duration but showed a significant main effect for photoreceptor‐directed stimulation (RMF‐ANOVA *F*_2,516_ = 69.37, *p* = 0.000, *η*_p_^2^ = 0.21) with the time to peak TEPD significantly delayed with melanopsin‐directed stimuli compared with the cone‐directed (Bonferroni's: 52.33 ± 17.16 ms, *p* = 0.008) or combination stimuli (Bonferroni's: 35.43 ± 14.25 ms, *p* = 0.03). There were no significant differences in the time to peak TEPD between cone‐directed and combination stimuli (Bonferroni's: 21.3 ± 16.12 ms, *p* = 0.08).

Our independent measurement of the melanopsin‐ and cone‐ photoreceptor inputs to the TEPD allowed evaluation of the process controlling how these photoreceptor signals are combined in the afferent pupil control pathway. The simplest description of the combined pupil response (Figure 2g, orange line) was a model (Figure 2g, black dashed line) incorporating a weighted linear addition of the melanopsin (Figure 2g, green line) and cone photoreceptor inputs (Figure 2g, grey line) where

(1) $$\begin{array}{l} {\text{TEPD combined}}_{\left(\mathrm{LMS}+\text{Melanopsin}\right)}\\ \;=c*{\text{TEPD}}_{\mathrm{LMS}}+m*{\text{TEPD}}_{\text{Melanopsin}}+S, \end{array}$$

and the cone weight, c = 0.42, the melanopsin weight, m = 0.66, and S is the scaling factor for post‐retinal summation. A Chi‐square goodness of fit test determined that the model was not significantly different from the measured combined pupil response (*χ* ^2^_1,359_ = 0.054, *p* = 0.70).

### 3.4 Melanopsin excitation produces a biphasic pupil response during decision‐making

**FIGURE 3:**
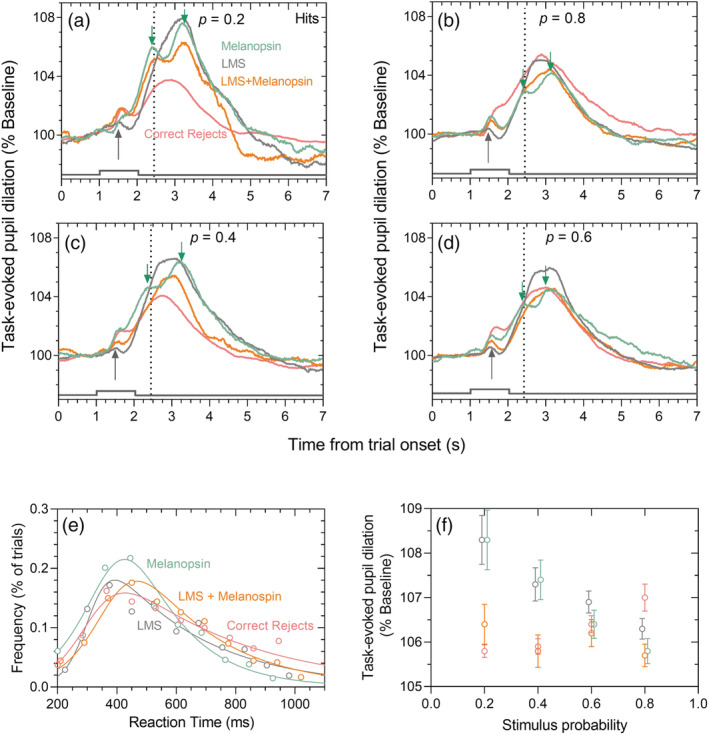
Photoreceptor‐directed pupil responses and reaction times during active attention in a visual discrimination task. Data (*n* = 5 observers) are coded green for melanopsin‐directed responses, grey for LMS‐cone‐directed responses, orange for the combined‐stimulus responses, and pink for correct rejects with non‐stimulus. (a)(b)(c)(d) task‐evoked pupil responses (% baseline pupil diameter) for hits and correct rejects for the four stimulus probabilities (*p* = 0.2, *p* = 0.4, *p* = 0.6, *p* = 0.8). The two conditions with higher and lower uncertainty are aligned in the left and right columns, respectively. Grey up‐arrows denote the pupil dilation to the auditory cue at stimulus onset; green down‐arrows denote the timing of the peaks of the biphasic dilation in response to melanopsin‐containing stimuli. The dashed vertical lines represent the average RT for all photoreceptor‐directed stimulus conditions. Pupil traces represent the average of 100 trials per stimulus condition per observer. (e) Visual choice RT distribution to the offset of threshold level photoreceptor‐directed stimuli for hits (i.e., with stimulus) and correct reject outcomes (i.e., no stimulus), pooled across all stimulus probabilities for all observers and trials, are described by their best‐fitting hyperbolic secant functions (solid lines). (f) Reaction time locked task‐evoked pupil dilation (TEPD) amplitudes (% baseline, average ± SEM) for hits and correct rejects

The preinformed stimulus probability was varied during the discrimination task to control the observer bias and unmask how decision certainty drives the TEPD (Figure 1b). A biphasic TEPD was discovered with hit‐responses for threshold level melanopsin‐activating stimuli (melanopsin and combined stimuli) at all four preinformed bias levels (Figure 3a–d, green and orange lines). The secondary dilation was always larger in amplitude than the first (two green downward arrows). In comparison, the TEPD was monophasic with hits for cone‐directed stimuli (grey lines) and for correct rejects (pink lines). Pupil recovery to baseline following decision responses across all stimulus probabilities, were longer with hit responses to melanopsin‐directed stimuli (2455 ± 257 ms; mean ± SEM) than cone‐directed (2110 ± 154 ms), combination stimuli (1865 ± 150 ms) or for correct rejects (2338 ± 419 ms). Independent of the stimulus presence or absence, the audio tone signalling trial onset caused a small amplitude dilation (1.63 ± 0.32%) occurring 456 ± 21 ms after stimulus onset (Figure 3a–d, grey upward arrows). This dilation was followed by a low amplitude transient constriction (0.42 ± 0.15%) at 675 ± 52 ms after which the pupil rapidly dilated during the decision‐making window and peaked (6.24 ± 1.32%) at 710 ± 64 ms after the observer's decision response (Figure 3a–d, dashed vertical lines).

### 3.5 The global arousal state defined by the TEPD is highest when uncertainty is greatest and independent of the afferent retinal pathway stimulation

To determine how intrinsic bias influences the RT and TEPD during decision responses to opsin‐initiated visual stimuli with a preinformed likelihood, we quantified the observer sensitivity (discriminability index, d′) and the response criterion (bias, c′) using signal detection theory according to the four response types. The RTs for hits were independent of stimulus probability but showed a significant main effect for photoreceptor‐directed stimulation (RMF‐ANOVA *F*_2,1324_ = 7.78, *p* = 0.000, *η*_p_^2^ = 0.01), with the RT significantly longer with combination stimuli (598 ± 18 ms) than to cone‐directed stimuli (554 ± 17 ms, Bonferroni's: 44.39 ± 12.00 ms, *p* = 0.001) but similar in response to melanopsin‐directed stimuli (587 ± 17 ms, Bonferroni's: 10.62 ± 11.29 ms, *p* = 1.00) (Figure 3e). This indicates that the combination stimuli were the least salient at threshold level. Consistent with this, all observers verbally reported that of the three stimuli, the combination‐directed stimulus was the most difficult to discriminate from the adapting background.

The TEPD amplitude for hits showed a significant main effect for the type of photoreceptor‐directed response (RMF‐ANOVA, *F*_2,1356_ = 9.68, *p* = 0.000, *η*_p_^2^ = 0.01), with combination stimuli producing significantly lower TEPD amplitudes than for the melanopsin‐ (Bonferroni's: 0.98 ± 0.27%, *p* = 0.001) or cone‐directed stimuli (Bonferroni's: 1.04 ± 0.26%, *p* = 0.000) at 20% and 40% probability, but not at 60% and 80% probability (Figure 3f). The hits TEPD amplitude also showed a significant main effect for stimulus probability (RMF‐ANOVA, *F*_3,1356_ = 6.80, *p* = 0.000, *η*_p_^2^ = 0.03). The lowest stimulus probability (20%) produced significantly larger hits TEPD amplitudes than the higher stimulus probabilities of 60% (Bonferroni's: 1.21 ± 0.40%, *p* = 0.02) and 80% (Bonferroni's: 1.70 ± 0.39%, *p* = 0.000) irrespective of the photoreceptor‐directed response. The interaction between type of photoreceptor‐directed response and stimulus probability did not show significant effect on the hits TEPD amplitudes.

The RTs for correct rejects (i.e., no stimulus) were independent of stimulus probability with the average RT at 614.82 ± 4.88 ms (Figure 3e). For correct rejects, the RT aligned TEPD amplitude showed a significant main effect of stimulus probability (RM‐ANOVA, *F*_3,2530_ = 5.49, *p* = 0.001, *η*_p_^2^ = 0.03). The TEPD amplitudes were significantly larger with the higher (80%) than lower stimulus probabilities (20% probability: Bonferroni's = 1.13 ± 0.32%, *p* = 0.003; 40% probability; Bonferroni's = 1.07 ± 0.34%, *p* = 0.01) (Figure 3f). It is therefore the preinformed likelihood that the probability of the choice is correct (i.e., the decision certainty) which drives the pupil dilation amplitude and not the expectation of a nontarget.

For missed responses (i.e., incorrect discrimination of stimuli), the TEPD amplitudes were independent of the opsin type and stimulus probability whereas RT showed a significant dependence on the type of photoreceptor‐directed response (RMF‐ANOVA, *F*_2,154_ = 5.89, *p* = 0.003, *η*_p_^2^ = 0.07). Missed responses to the combination stimuli produced significantly longer RTs than for melanopsin‐directed (Bonferroni's: 106.46 ± 34.95 ms, *p* = 0.01) or cone‐directed stimuli (Bonferroni's: 119.03 ± 38.98 ms, *p* = 0.01) with no significant differences in RTs for cone‐ and melanopsin‐directed stimuli (Bonferroni's: 12.57 ± 40.22 ms, *p* = 1.00). While the visual RT to missed responses were ~175 ms longer than their corresponding hits RTs, the TEPD amplitudes on false alarms were ~2% larger than on the hits responses. With false alarms, both the RT and TEPD amplitudes were independent of the stimulus probability. No false alarm responses were recorded in our sample for melanopsin‐directed stimuli.

**FIGURE 4:**
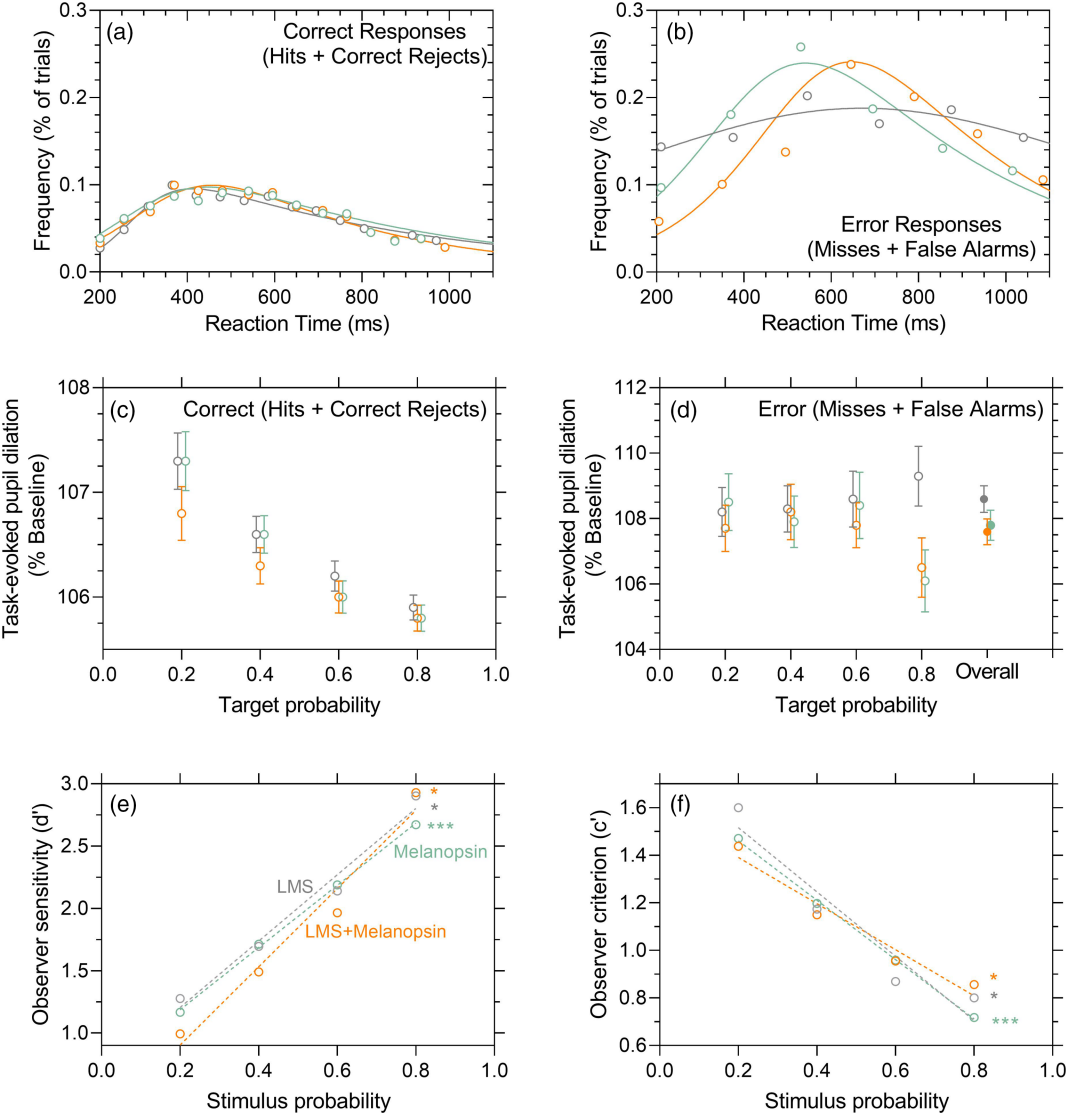
Signal detection theory (SDT) analysis of the task‐evoked pupil dilation (TEPD) amplitude and choice reaction time (RT) as per the correctness of the decision. Data (*n* = 5 observers) are coded green for melanopsin‐directed responses, grey for LMS‐cone‐directed responses and orange for the combined‐stimulus responses. (a) Visual choice RT distribution for correct responses (hits + correct rejects) pooled across all the four stimulus probabilities (*p* = 0.2, *p* = 0.4, *p* = 0.6, *p* = 0.8) for all observers. (b) Visual choice RT distributions with error responses (misses + false alarms) pooled across all stimulus probabilities. (c) Reaction time locked TEPD amplitudes (% baseline, average ± SEM) for correct responses and (d) error responses. In panels (c) and (d), data represent the mean ± SEM of pooled data from five observers. Rightmost data points in panel (d) (overall, solid circles) represent the average data pooled across all stimulus probabilities. (e) SDT analysis of the observer sensitivity (d′) and (f) observer criterion (c′) to discrimination of photoreceptor‐directed stimuli as a function of stimulus probability (*n* = 5 observers). The dashed lines are the best fitting linear regression lines for each photoreceptor‐directed stimulation condition. Asterisks represent p‐values, *p < 0.05; ***p < 0.001 for slopes of regression lines significantly different from zero

The RT's for correct responses (hits + correct rejects) were independent of the target (stimulus or no‐stimulus) probability and photoreceptor‐directed stimulation (Figure 4a). The TEPD amplitudes were independent of photoreceptor directed stimulation but showed a significant main effect of target probability (RMF ANOVA, *F*_3,3209_ = 23.18, *p* = 0.000, *η*_p_^2^ = 0.021) (Figure 4c). The TEPD amplitude with correct responses was significantly larger when the target probability was lower (20%) compared with the higher target probabilities (40% probability: Bonferroni's = 0.57 ± 0.19%, *p* = 0.03; 60% probability: Bonferroni's = 1.07 ± 0.18%, *p* = 0.000; 80% probability: Bonferroni's = 1.27 ± 0.17%, *p* = 0.000). The RTs and TEPD amplitudes with error responses (misses + false alarms; Figure 4b,d) were independent of stimulus probability and photoreceptor‐directed stimulation. Error responses resulted in significantly longer choice RTs (Figure 4b) than with correct responses (Figure 4a) for all photoreceptor‐directed stimuli (RT difference melanopsin = 84 ± 27 ms, *t*_154_ = 0.48, *p* = 0.02; RT difference cone = 94 ± 25 ms, *t*_187_ = 3.77, *p* = 0.000; RT difference combination = 123 ± 24 ms, *t*_188_ = 5.06, *p* = 0.000). For lower target probability, all photoreceptor‐directed stimuli produced a significantly larger TEPD amplitude for a correct response (RMF‐ANOVA, *F*_3,2846_ = 9.56, *p* = 0.001, partial *ɳ* ^2^ = 0.007) (Figure 4c); the TEPD however is not significantly dependent on target probability for an error response (RMF‐ANOVA, *F*F_3,158_ = 0.99, *p* = 0.40, partial *ɳ* ^2^ = 0.03) (Figure 4d). The TEPD amplitudes however were significantly larger with error responses (Figure 4d) than correct responses (Figure 4c) for the combination‐directed stimuli (TEPD difference = 1.02 ± 0.50%, *t*_206_ = 2.04, *p* = 0.04), but not melanopsin or cone‐directed stimulation. This, together with a larger proportion of error responses for the combination stimuli suggests a higher uncertainty when discriminating combination stimuli and that melanopsin and cone inputs to the TEPD may show differential interactions that are dependent on discrimination accuracy.

Our SDT analysis is based on the number of trials giving an outcome, thus d′ and c′ are the same for both pupil and RT data (Figure 4e,f). Observer sensitivity (d′; Figure 4e) increased with increasing stimulus probability for all three threshold level stimuli (melanopsin‐directed: *r* ^2^ = 0.99, *F*_1,2_ = 1953, *p* = 0.000, cone‐directed: *r* ^2^ = 0.98, *F*_1,2_ = 83.87, *p* = 0.01 and combination stimuli: *r* ^2^ = 0.97, *F*_1,2_ = 58.38, *p* = 0.02). The observer criterion (c′; Figure 4f) decreased with increasing stimulus probability (melanopsin‐directed: *r* ^2^ = 0.99, *F*_1,2_ = 1953, *p* = 0.000; cone‐directed: *r* ^2^ = 0.92, *F*_1,2_ = 22.30, *p* = 0.04; combination stimuli: *r* ^2^ = 0.95, *F*_1,2_ = 42.12, *p* = 0.02). Positive c′ values reflect the adoption of a strict (conservative) criterion for all threshold level stimuli and the decrease in c′ with corresponding increase in d′ at higher stimulus probabilities is due to better decision certainty produced by priming rather than the response to photoreceptor‐directed stimulus.

The effect of the interaction between stimulus contrast and decision certainty on the TEPD amplitude was determined by comparing the average pupil responses in the supra‐threshold (detection experiment) and threshold level (discrimination experiment) photoreceptor‐directed conditions (Figure 2e vs. Figure 3f). When all three types of photoreceptor‐directed stimuli were merged for the analysis, the TEPD amplitudes to supra‐threshold (100% probability: 7.57 ± 0.15%) and threshold level stimuli (20% probability: 6.92 ± 0.45%) were not significantly different (*t*_1132_ = 0.96, *p* = 0.06) whereas TEPD amplitudes to supra‐threshold stimuli (100% probability) were significantly larger than to threshold level stimuli at 80% probability (3.89 ± 0.50%) (*t*_1841_ = 7.57, *p* = 0.000). We infer that stimulus contrast, which affects saliency through a bottom‐up process, may interact with decision‐uncertainty, a top‐down process, to influence the TEPD amplitude during decision‐making for all photoreceptor‐directed inputs.

## 4 DISCUSSION

The TEPD amplitude that marks an observer's decision during active covert attention is determined through summation of the melanopsin and cone photoreceptor (L‐, M‐, and S‐cone) inputs to the afferent pupil control pathway (Figure 2b,e). When independently controlled, the melanopsin‐ and cone‐directed stimuli produce similar TEPD amplitudes (Figure 2e), which suggests that the melanopsin‐directed photostimulation did not boost the global arousal state more than that observed with cone‐directed photostimulation. The later time‐to peak TEPD for melanopsin‐ than cone‐directed stimulus (Figure 2f) is due to the longer melanopsin integration time. Together, the combined melanopsin and cone‐directed stimulation produces a significantly larger TEPD amplitude that is modelled by their linear addition with a greater melanopsin weighting (Figure 2g). This indicates the intrinsic melanopsin signal has higher gain in the pupil pathway during active covert attention and may explain previous observations (Chellappa et al., 2014; Vandewalle et al., 2007) that amygdala, pulvinar and superior colliculus activity increases with melanopsin‐enriched lights that residually stimulate the cone pathways compared with the lower arousal responses observed with cone‐enriched lights. Therefore, the executive processes controlling the decision and the top‐down modulation of the efferent pupil signals driving the TEPD will both require integration windows longer than the difference in the transmission times of the melanopsin and cone signals to evaluate all available visual information.

The photopic visual RT was ~115 ms longer to melanopsin‐directed (~575 ms) than cone‐directed stimuli (Figure 2d). For comparison, rod and cone RT's measured under the same mesopic adaptation conditions differ only by ~20 ms (Cao et al., 2007). Evidence of the slower melanopsin temporal response from physiological recordings of single cells (Dacey et al., 2005; Do et al., 2009) indicates these latency differences are already present in the retina. Lights with higher melanopsin excitation are also perceived to be of longer duration than cone‐directed lights (Yang et al., 2018). Both the combination and cone‐directed RTs were similar (Figure 2d), as expected, because the faster cone‐mediated information is processed first in simple RT experiments (Cao et al., 2007). After RT alignment, the peak melanopsin‐directed TEPD remained significantly delayed compared with the timing of the cone‐directed TEPD (~64 ms, Figure 2f) whereas the TEPD amplitudes were similar (Figure 2e). We infer that direct ipRGC projections to decision‐making brain areas (Chellappa et al., 2011; Vandewalle et al., 2007; Viola et al., 2008) that can modulate phasic arousal can also conserve the latency differences that exist between the melanopsin and cone signals in the retina.

Active attention produced a smaller baseline pupil diameters than the passive attention conditions (Figure 2c) in accordance with focused‐states involving active engagement (Unsworth & Robison, 2016). The motor response, depending on the task difficulty, contributes to the TEPD (Privitera et al., 2010). If the TEPDs were only due to the motor component related to the task, no effect of photoreceptor type would have been observed, but this was not the case. While we acknowledge that a requirement of a motor response to the presence of a visual stimulus produces an initial pupil dilation (denoted by the grey upward arrow in Figure 3a), the differences in the composite TEPDs between the three photoreceptor‐directed stimulus types arise largely due to the direct effect of the differential photoreceptor contributions to the visual decision‐making pathway.

By fixing the predecision intrinsic bias to control the phasic arousal level (Figures 3 and 4), we constrained a known source of variation in the TEPD amplitude (de Gee et al., 2017). Doing so, our results demonstrate that stimulus contrast, which determines saliency through a bottom‐up process, interacts with decision‐uncertainty, a top‐down process, to control the TEPD amplitude during decision‐making for all photoreceptor‐directed inputs. The TEPD amplitudes for low contrast stimuli with low decision certainty (20% probability) are similar to higher contrast stimuli with high decision certainty (100% probability) (Figure 3a vs. Figure 2e). That the TEPD amplitudes during detection of supra‐threshold stimuli were significantly (~4%) larger than with the correct discrimination of threshold level stimuli (80% probability), indicates that contrast makes an independent contribution to the TEPD. These findings are in accord with larger TEPD amplitudes observed during visual discrimination (66% stimulus probability) of higher than lower contrast stimuli in monkeys (Wang et al., 2014). With supra‐threshold audio stimuli there is also evidence for an inverse relationship between TEPD amplitude and stimulus probability (Qiyuan et al., 1985). Thus, for a low contrast stimulus, higher decision uncertainty in visual discrimination could compliment the phasic arousal state independent of the afferent input.

When there is low certainty of correctly discriminating a target, we show that even the absence of a stimulus produces a large TEPD amplitude (Figure 3f). For all measured target probabilities (20% to 80%), the TEPD amplitudes with hits for melanopsin‐ and cone‐directed threshold level stimuli and correct rejects (Figure 3f) decreased with increasing target probability. In the absence of reward or penalty, as in this study, an observer's conscious state of attention (top‐down control) influences the TEPD more than the attention capturing aspects of the stimulus (bottom‐up control). The top‐down manipulation of task difficulty by adding spatial structure, colour, sound and/or shape as secondary tasks can lead to decreased visuospatial awareness and greater task engagement which is indexed by larger TEPD amplitudes (Lisi et al., 2015). When uncertainty is high (20% stimulus probability), the TEPD begins ~900 ms before onset of the low contrast photoreceptor‐directed stimulus (Figure 3a). Consistent with this, perceptual decisions are known to occur ahead of the overt response as evidenced by the persistent, frequency sensitive magnetoencephalography activity in motor cortical areas during visual motion detection tasks (Donner et al., 2009) and the early initiation of the pupil dilation in speeded RT tasks (Richer et al., 1983). Taken together, the mechanism controlling TEPD is not dependent on the mode of sensory stimulation (e.g., auditory and visual) or as demonstrated here, the visual opsin, and so the TEPD amplitude for threshold level stimuli is then determined jointly by the preparatory process for a motor response and the uncertainty‐driven decisional inputs.

The differences in the shape, amplitude, and timing of the photoreceptor‐directed task‐evoked pupil responses open the way for its application as a marker of neuromodulatory activities during visual decision‐making, which currently relies on electrophysiological measures. Detection of melanopsin‐directed supra‐threshold stimuli (Figure 2b) and hits for threshold level melanopsin‐directed stimuli (Figure 3a–d) caused a signature biphasic TEPD (green downward arrows); the first dilation (~4%) occurred ~300 ms following stimulus offset and was of lower amplitude than the second dilation (~5%) occurring ~1200 ms later. This biphasic TEPD was absent with hits for cone‐directed visual stimuli and correct rejects suggesting that the biphasic TEPD is an attribute of the melanopsin pathway. While the physiological origin of the first dilation is still to be determined, there are direct ipRGC projections to both the suprachiasmatic nucleus (SCN) and olivary pretectal nucleus (Berson et al., 2002; Hannibal et al., 2014). The activity of the mouse SCN increases in response to lights with higher melanopsin excitation in the wild‐type but not melanopsin‐deficient mice (Pilorz et al., 2016) and this increased SCN activity might contribute to a faster first pupil dilation with melanopsin than cone‐directed light due to the arousal promoting function of the SCN (Aston‐Jones et al., 2001). The second dilation, however, is common to all photoreceptor‐directed stimulations and is thought to result from the attention triggered sympathetic activation of the pupillary dilator muscles (Steinhauer & Hakerem, 1992).

With higher decision certainty, melanopsin‐ and cone‐directed inputs to the TEPD combine through a simple weighted linear addition (Figure 2g), consistent with afferent pupil light responses to melanopsin‐ and cone‐directed light pulses combining additively in ipRGCs (Zele et al., 2019a) during passive attention. A different integration pathway is however evident for the threshold level stimuli. Although the overall signal to noise ratio is low for all three photoreceptor‐directed threshold level stimuli, a lower TEPD amplitude was observed in response to combination stimuli than with either the melanopsin‐ or cone‐directed stimuli (Figure 3a). This lower amplitude TEPD with combination stimuli might be due to destructive interference between the intrinsic melanopsin and cone signals as evidenced in the white noise electroretinogram (Adhikari et al., 2019) and involve nonlinear cone inputs to ipRGCs (Barrionuevo et al., 2018). That the longer latency of the intrinsic melanopsin response than the cone response (Dacey et al., 2005; Do et al., 2009) manifests only in the supra‐threshold stimulus conditions (Experiment 1, Figure 2d) and not for the threshold conditions (Experiment 2, Figure 3e) suggests there exists a differential interaction between stimulus contrast and decision uncertainty for different photoreceptor pathways. It is possible that these differential interactions result in different types of summation between melanopsin and cone signals in the supra‐threshold and threshold conditions (Figures 2g and 3f).

Hit responses to the photoreceptor‐directed threshold level stimuli produced similar TEPD amplitudes as with the correct rejects. Therefore, the absence of a reward for correct discriminations (Colizoli et al., 2018; Urai et al., 2017) might prevent larger TEPD amplitudes for hits than for misses, as previously reported for some visual detection tasks (Hakerem & Sutton, 1966; Privitera et al., 2010). The low salience, threshold level stimuli raised the decision uncertainty and forced observers to set a strict (conservative) criterion during decision‐making (Figure 4f) that resulted in the longer choice RTs and smaller TEPD amplitudes than with the supra‐threshold stimuli. It is however, an incorrect (error) decision that leads to the largest TEPD amplitudes and longest visual RTs, independent of the photoreceptor‐directed stimulations (Figure 4b,d). Larger TEPD amplitudes with error responses than with correct responses is consistent with methodologies applying equiprobability targets to compare TEPDs during decision formation and after reward‐linked feedback (Colizoli et al., 2018; Urai et al., 2017) but contrasts with the findings of de Gee et al. (2014), who report similar TEPD amplitudes between correct choices and errors but larger TEPD amplitudes for yes compared with no choices. In that study, their paradigm was not designed to independently modulate the intrinsic bias in the observers as we did here. Instead, their observer bias was estimated post‐hoc to the behavioural response (de Gee et al., 2014), and so their larger TEPD amplitudes could be driven by the missed responses from conservative subjects. The decreasing observer bias (c′; Figure 4f) and TEPD amplitude (Figure 3f) with increasing stimulus probability indicates direct relationship between phasic arousal and decision bias, likely due to the locking of the predecisional intrinsic bias.

## 5 CONCLUSIONS

We observed that decision‐making centres during active covert attention tasks can utilise visual information mediated via either or both the melanopsin and cone pathways. Phasic arousal responses as marked using the TEPD amplitude are similar for both melanopsin and cone‐enriched lights, with the melanopsin response characterised by a biphasic dilation. With supra‐threshold stimuli, the amplitude and timing of the TEPD depends on the type of photoreceptor‐directed stimulation and the high decision certainty. In contrast, decision uncertainty is the primary driver of phasic arousal with threshold level stimuli, independent of the level of melanopsin stimulation in the light.

## CONFLICT OF INTEREST

The authors declare no competing financial interests.

## AUTHOR CONTRIBUTIONS

AJZ and BF envisaged the experiments. SG, BF, PA, and AJZ designed the experiments. SG performed the research. BF and AJZ supervised data collection. SG and AJZ analysed data. AJZ and SG wrote the paper. BF and PA reviewed and edited the manuscript.

### PEER REVIEW

The peer review history for this article is available at https://publons.com/publon/10.1111/ejn.15659.

## Data Availability

The findings of this study are available within the article and from the corresponding author, upon reasonable request.
